# Patient autonomy in a digitalized world: supporting patients’ autonomous choice

**DOI:** 10.3325/cmj.2016.57.80

**Published:** 2016-02

**Authors:** Øystein Ringstad

**Affiliations:** Department for Health and Social Studies, Østfold University College, Halden, Norway

Today, huge amounts of knowledge and information are easily accessible on the internet to anyone who can operate a computer (1,2). We might expect this development to enable a shift of power from the physician to the patient and thus enhanced patient autonomy. Some even anticipate a new role for physicians as assistants who fulfill the wishes of fully updated patients knowing more about their disease than the doctor (sometimes referred to as “internet patients”). On the other hand, skeptics might claim that with all that information available but without proper quality assurance, patients will more than ever need experts' competence to protect them from serious misunderstandings and futile or harmful treatments.

Both of these crude and polarized positions are probably true, but need to be further reflected upon. On the one hand, it is important to counter misinformation and misunderstandings stemming from uncritical distribution and reading of biomedical information. On the other hand, trustworthy communication of biomedical knowledge may contribute to the empowering of patients (3). In this paper, I will discuss how these two polarities can be balanced in the light of the bioethical principle of respect for patient autonomy. I will argue that there may be good reasons why the doctor should at the same time acknowledge and be critical of the patient’s search for diagnoses and treatments far frommainstream medical practice or the patient’s personal experience. Being aware of such reasons, the doctor might be better prepared to respect the patient’s autonomy.

## Patient autonomy

Autonomy is a complex and multifaceted concept. Two of its core connotations are “self-determination” and “self-governance,” referring to the processes by which persons make their own decisions and control their own lives. In the physician’s encounter with “the internet patient” there are two relevant aspects: “autonomous choice” and “personal autonomy.” When patients make choices about specific health care interventions, we speak about autonomous choice (4), while “personal autonomy” focuses on other aspects of being an autonomous person that are broader than just making autonomous choices (5). Autonomy is a question of dealing with the disease in the everyday life, not only making decisions about specific matters (6,7). This paper discusses the autonomous choice aspect of patient autonomy.

## Autonomous choice

Autonomous choice, thoroughly described by Beauchamp and Childress (4), has become the predominant conception of patient autonomy. According to their analysis, the procedure of obtaining informed consent is an instance of giving patient an opportunity of autonomous choice in the medical consultation. If a choice or decision is to be considered autonomous, it should be free from the controlling influence of others, for instance the doctor. Moreover, it should also be made with a substantial degree of understanding and with an intention to choose. Substantial understanding means that the patient has understood the relevant information, not only theoretically, but also that he or she appreciates the impact that a certain decision might have on his or her body or life.

## The medical consultation

During a traditional medical consultation, the patient will present the chief complaint, but the physician will make the diagnosis, perform or order the relevant tests, and recommend the treatment. However, patients’ opinions may often be a suitable basis for the discussion about test results, treatment recommendations, and prognostic expectations. The aim of informed consent procedures is to promote autonomous choice by disclosing sufficient information about the recommended interventions and let the patient consent or refuse without the undue influence of others. However, the patient may also offer information and questions from sources that the doctor is less acquainted with or does not consider relevant. The sources on the internet provide explanations for the patient’s symptoms and propose laboratory investigations and treatments. Moreover, there are also available discussions, blogs, and videos presenting other patients’ experiences. However, the patients sometimes do not possess the critical skills to determine whether the purpose of these web-sites is purely commercial and whether the data available are based on research or solely on patients’ personal views.

Doctors may react in different ways on this intrusion into the conventional consultation agenda. They could feel that the patient challenges their professional control and authority or expresses distrust and dissatisfaction with their handling of the case. There are also more rational reasons to be skeptical about such initiatives from the patient. In a busy clinical practice, it may seem a waste of time to discuss patients’ sometimes far-fetched, irrelevant, and unreliable information from unsystematic and uncritical searches on the internet. The patient may also think that the doctor confirms his misunderstandings by allocating time to discuss them.

As a medical doctor, I think it is important to be aware of our immediate reactions and give them a second thought. Although they may sometimes be justified, we should also be able to see such initiatives as a sign of patients’ commitment to take control of their own health. This view may even be crucial, if the patient has found important information that the doctor did not know about. Anyhow, we should respond to patients’ wish to make informed and rational choices about their health. As doctors, we should and we can support and help them in making autonomous choices.

## The autonomous choice of “the internet patient”

In informed consent procedures the doctor normally chooses which interventions to address. Doctors must then base their information and recommendations on well-established medical knowledge and guidelines. However, when “the internet patient” wants to set the agenda and discuss suggestions based on more unfamiliar sources, doctors should be prepared to address such questions, too. Still, the same quality criteria should be used when patients seek answers on the internet as when the doctor makes the recommendations.

One conclusion might simply be to warn the patient against treatments that are not supported by established practices and guidelines. This may indeed be both a safe and time-saving approach, avoiding further discussion. Still, it may not fulfill all patients’ need for information and understanding as preconditions for their autonomous choice. Some may even see such adherence to guidelines as a sign that doctors are too submissive to medical authority, expecting us to explain the rationale of evidence based medicine more in detail. Here I will suggest three issues that may be difficult for lay people to understand properly without professional advice.

## Reliability, relevance, and individual approach as key concepts

The first issue is the reliability of the information the patient brings into the discussion. It is important to understand that clinical decisions require information of much greater reliability than other types of decisions. Our question is whether the information is reliable as a basis for performing the suggested diagnostic or therapeutic intervention on this particular patient. In this case, the doctor needs to acquaint the patient with the hierarchy of evidence, in which case reports are considered the lowest level of evidence and systematic reviews of randomized controlled trials are preferred as basis for clinical guidelines (8,9).

The next issue is whether the information is relevant to this particular patient’s case. Patients probably tend to underestimate the importance of this concern, regarding all cases with similar symptoms or similar diagnoses as one group, generalizing results or experiences to all members of the group without considering relevant differences. Clinical work means to carefully evaluate the relevance of general knowledge to the individual case, based on the necessary diagnostic tests and correct classification according to stage and severity of the disease (9).

The third question is how the individual patient’s goals and preferences relate to the intervention in question. One important distinction is between interventions that modify the disease process, increasing the probability of a favorable outcome, and interventions that only relieve symptoms and improve the quality of life. This distinction may not always be clearly communicated in the published information, but it is nevertheless essential that patients understand it to be able to make informed and rational choices (4).

Taking patients’ questions seriously may enhance their autonomy. By being willing to discuss issues stemming from patients’ own search for information the doctor may contribute to an improved communication about biomedical knowledge, acknowledging patients’ efforts and empowering patients by helping them to interpret the meaning of such information in their individual case (3). Sometimes they ask about simple measures that are easy to support. Other suggestions cannot be supported for more complex medical reasons. Being transparent about our professional reasoning may contribute to the patient’s autonomous choice based on proper understanding of the available information.
